# Predictors of Time to Recovery in Infants with Probable Serious Bacterial Infection

**DOI:** 10.1371/journal.pone.0124594

**Published:** 2015-04-24

**Authors:** Prashant Singh, Nitya Wadhwa, Rakesh Lodha, Halvor Sommerfelt, Satinder Aneja, Uma Chandra Mouli Natchu, Jagdish Chandra, Bimbadhar Rath, Vinod Kumar Sharma, Mohini Kumari, Savita Saini, Sushil Kumar Kabra, Shinjini Bhatnagar, Tor A Strand

**Affiliations:** 1 Translational Health Science and Technology Institute, Gurgaon, India; 2 Department of Pediatrics, All India Institute of Medical Sciences, New Delhi, India; 3 Centre for Intervention Science in Maternal and Child Health (CISMAC), Centre for International Health, University of Bergen, Bergen, Norway; 4 Department of International Public Health, Norwegian Institute of Public Health, Oslo, Norway; 5 Department of Pediatrics, Lady Hardinge Medical College and Kalawati Saran Children’s Hospital, New Delhi, India; 6 Department of Pediatrics, Deen Dayal Upadhyay Hospital, New Delhi, India; 7 Division of Medical Services, Innlandet Hospital Trust, Lillehammer, Norway; University College London, UNITED KINGDOM

## Abstract

**Introduction:**

Serious bacterial infections continue to be an important cause of death and illness among infants in developing countries. Time to recovery could be considered a surrogate marker of severity of the infection. We therefore aimed to identify clinical and laboratory predictors of time to recovery in infants with probable serious bacterial infection (PSBI).

**Methods:**

We used the dataset of 700 infants (7-120 days) enrolled in a randomised controlled trial in India in which 10mg of oral zinc or placebo was given to infants with PSBI. PSBI was defined as signs/symptoms of possible serious bacterial infection along with baseline C-reactive protein(CRP) level >12mg/L. Time to recovery was defined as time from enrolment to the end of a 2-day period with no symptoms/signs of PSBI and daily weight gain of at least 10g over 2 succesive days on exclusive oral feeding. Cox proportional hazard regression was used to measure the associations between relevant variables and time to recovery.

**Results:**

Infants who were formula fed prior to illness episode had 33% longer time to recovery (HR-0.67, 95%CI-0.52, 0.87) than those who were not. Being underweight (HR-0.84, 95%CI-0.70, 0.99), lethargic (HR-0.77, 95%CI-0.62, 0.96) and irritable (HR-0.81, 95%CI-0.66, 0.99) were independent predictors of time to recovery. Baseline CRP was significantly associated with time to recovery (P<0.001), higher CRP was associated with longer time to recovery and this association was nearly linear.

**Conclusion:**

Simple clinical and laboratory parameters such as formula feeding prior to the illness, being underweight, lethargic, irritable and having elevated CRP levels could be used for early identification of infants with PSBI at risk for protracted illness and could guide prompt referral to higher centers in resource limited settings. This also provides prognostic information to clinicians and family as longer recovery time has economic and social implications on the family in our setting.

**Trial Registration:**

ClinicalTrials.gov NCT00347386

## Introduction

Every year, about 6.3 million children under 5 years of age die globally. Of these, about 2.8 million die in their first month of life [1]. One-fifth of these neonatal deaths occur because of serious bacterial infections like sepsis and pneumonia [1]. About 0.8 million neonates die every year in India and this constitutes more than a quarter of all neonatal deaths worldwide.[2] Serious bacterial infections account for more than a quarter of the neonatal deaths in India and serious bacterial infections continue to be an important cause of death in infants beyond the neonatal period [3].

Despite advances in antimicrobial treatment and increased availability of health facilities, the outcome in infants with serious bacterial infection remains poor [4]. Many infants with serious bacterial infection never reach treatment facilities and the case fatality for those who do, range from 13% to 69% [4]. Recovery time can be considered a surrogate marker of severity and extended hospital stay imposes an economic burden on both family and limited health budgets in developing countries.

Many studies have identified markers of poor prognosis in neonates with sepsis, [5–7] however, there is no data on predictors of time to recovery in infants with serious bacterial illness. Early identification of such predictors can facilitate an aggressive management at the outset and identify children that should receive special attention. We conducted a secondary analysis using a dataset of 700 infants enrolled in randomised controlled trial on the efficacy of zinc given as an adjunct to standard antibiotics for the treatment of young infants with probable serious bacterial infection (PSBI), to identify clinical and laboratory predictors of time to recovery [8].

## Materials and Methods

### Study site

The study was conducted at three tertiary hospitals in New Delhi, India (All India Institute of Medical Sciences, Deen Dayal Upadhyay Hospital and Kalawati Saran Children’s Hospital) from July 2005 till December 2008. Ethics committee of these three hospitals and World Health Organization’s Ethics review committee approved the study protocol of the parent study. This study is a result of secondary analysis of the same data collected for the parent study. No new data was collected for this study. There was no patient interaction for this secondary analysis. Written informed consent was obtained from guardians of infants for collection of data for the parent study. Patients’ information was anonymized and de-identified prior to this secondary analysis. Infants aged 7–120 days were screened in the emergency departments for clinical symptoms and/or signs of possible serious bacterial infection adapted from the Integrated Management of Neonatal and Childhood Illnesses (IMNCI) strategy [8, 9].

### Study design

Infants with any one or more signs/symptoms of possible serious bacterial infection adapted from IMNCI criteria along with a serum C-reactive protein (CRP) concentration ≥12mg/L, measured semi-quantitatively with a latex agglutination assay, were defined to have PSBI and enrolled if their guardians gave their written informed consent [8]. With computer-generated sequences, we randomly assigned infants in permuted blocks of six, stratified by whether patients were underweight or had diarrhea at enrolment, to receive either 10 mg of zinc or placebo orally every day in addition to standard antibiotic treatment until recovery or 21 days (504 hours) whichever was earlier. Time to overall recovery was defined *a priori* as time from enrolment to the end of a 2-day period with no symptoms or signs of PSBI along with daily weight gain of at least 10g over 2 successive days, on exclusive oral feeding. The days of weight gain were counted only when all the symptoms/signs of PSBI had resolved.

Infants were treated with antibiotics and we provided supportive measures according to a standardized protocol. Once admitted, relevant clinical features were monitored by the study team every 6 h, or more often if clinically indicated, until the recovery or end of day 21, whichever was earlier. If the child had not recovered till three weeks from admission, he/she was no longer followed by the study team but they were managed by the clinical team until their discharge from the hospital. Respiratory rate was considered elevated if it was ≥ 60 breaths/min in infants younger than 2 months, and if≥ 50 breaths/min in those that were 2 months or older. If an infant was found to have an elevated respiratory rate, it was counted again and the second count was recorded. Weights of naked infants were registered at the time of randomization and then daily until recovery or end of day 21, whichever was earlier.

### Laboratory procedures

Blood samples for serum zinc, CRP and procalcitonin (PCT) levels were collected by the study physicians at enrolment, 72 hours thereafter, and at recovery. Using a standard protocol, sera was separated at the study site and transported to the central laboratory at AIIMS where it was stored at -20°C. Serum zinc was measured with a flame furnace atomic absorption spectrophotometer (GBC Avanta, Dandenong, VIC, Australia) using standard procedures. Seronorm (Sero AS, Billingstad, Norway) was used as the external reference standard after every tenth sample. CRP was measured quantitatively with a commercial enzyme linked immunosorbent assay (ELISA) (Biocheck, Foster City, CA, USA) and procalcitonin (PCT) using a chemi-immunoluminescence assay (BRAHMS, Hennigsdorf, Germany). For CRP, a pooled plasma of known concentration was used for quality assurance. The PCT kits had negative and positive controls which were used with each batch of samples. These assays were performed by a trained technical officer. Blood cultures were drawn at the time of enrolment along with other labs including haemoglobin and total leucocyte counts. These three laboratory values along with the baseline serum zinc, CRP and PCT measurements were included in our analysis.

### Quality assurance and standardization

Study physicians who were medical graduates evaluated the infant at the time of screening, enrolment, follow up and outcome assessment. We had three study physicians at each of the study sites providing coverage round the clock. Each site had a pediatrician as supervisor. He or she oversaw at least one daily monitoring conducted by each study physician.

The site supervisors were provided with training in patient assessment and outcomes, according to a standard protocol. The same protocol was followed across the three hospitals to ensure uniformity. The supervisors in turn trained the study physicians in collecting clinical information and examining the infant for eligibility, follow up and determination of study end points. The study physicians were also trained for seeking written informed consent from the parents/guardians of the participants. Besides the initial training, we had standardization exercises on clinical outcomes and laboratory procedures every 15 days within each site and once a month across the three sites to minimize intra- and inter-observer variability.

### Data collection

All forms were checked manually by supervisors and physicians for completeness and consistency. The data was then double entered in Microsoft Access (2007) with incorporated logic, range and consistency checks. If errors were detected, the forms were returned to the study site for correction the next working day. Weight for age z-scores were calculated using the lambda-mu-sigma (LMS) values obtained from Center for Disease Control (CDC) growth charts [10].

### Statistical Analysis

Statistical analyses were done using Stata, version 11.0 (StataCorp, College Station, TX, USA).

Cox proportional hazard regression was used to estimate the associations between potential predictors and time to recovery. We used CRP, procalcitonin, hemoglobin, total leukocyte count and serum zinc levels as continuous variables for the analysis. Based on the results of unadjusted regression models, all variables with P <0.2 were included in a multivariable Cox regression model. Variables that were no longer significant at this cut-off were removed and each of the variables that were excluded after the first assessment was added back one at a time. In the final model we only retained variables that were significant at P<0.05. This manual variable selection procedure was confirmed with automatic backward and forward stepwise selection using the Stata-command “sw” (step wise regression). A number of possible interactions between the independent variables were also assessed (irritability x lethargy, lethargy x poor sucking at breast, formula milk feeding x weight for age < -2 z, weight for age < -2z x poor sucking at breast). The correlation of CRP values (mg/L) and time to recovery (in hours) was expressed by the Spearman rank-order correlation coefficient.

## Results

Seven hundred (245 female) infants were included in the study with a mean (standard deviation, SD) age of 54.5 (29.9) days. The median (IQR) time to recovery was 133 (100.5, 175) hours.

The baseline clinical, anthropometric and laboratory details are shown in Table 1. About one-third of the infants were irritable and/or lethargic and/or had diarrhea at the time of admission. More than half of the infants had cough and a similar proportion had fast breathing on physical examination. 375 (53.6%) infants in our study were underweight. The enrolled subjects had median (IQR) CRP and procalcitonin concentrations of 33.7(18.4, 61.2) mg/L and 0.99(0.44, 4.8) μg/L, respectively. Half (50.3%) were randomized to receive 10 mg elemental zinc every day.

**Table 1:** Baseline clinical, anthropometric and laboratory details of 7–120 day old infants with probable serious bacterial infection.

	N = 700
**Demographic details**	
Age (days); mean [SD]	54.5 (29.9)
Female	245 (35%)
**Clinical history**	
Irritability	230 (32.9%)
Excessive Cry	275 (39.3%)
Diarrhea	256 (36.6%)
Blood in stools	15 (2.1%)
Vomiting	141 (20.1%)
Cough	358 (51.1%)
Rapid breathing	377 (53.9%)
Convulsions	59 (8.4%)
Edema	05 (0.7%)
Poor oral acceptance	288 (40.3%)
History of breast milk feeding prior to the episode	569 (81.3%)
History of cow milk feeding prior to the episode	178 (25.4%)
History of formula milk feeding prior to the episode	112 (16%)
History of antibiotic treatment in the current episode	141 (20.1%)
**Anthropometry**	
Weight for age less than -2z	375 (53.6%)
**Physical examination**	
Fever^a^	279 (39.9%)
Lethargy on Physical exam	208 (29.7%)
Poor sucking at breast	259 (37%)
Fast breathing ^b^	351 (50.1%)
Crepitations	275 (39.3%)
Grunting	42 (6%)
Wheeze	88 (12.6%)
Cyanosis	03 (0.4%)
Capillary refill time > 3 seconds	08 (1.1%)
Abdominal distention	36 (5.1%)
Oral thrush	16 (2.3%)
Some dehydration	63 (9%)
Bulging fontanel	18 (2.6%)
**Laboratory parameters**	
Hemoglobin; g/dL	11.2±3
TLC; per μL of blood	11,000 (8000–15,510)
Serum CRP ^c^; mg/L	33.7 (18.4–61.2)
Serum PCT ^c^; ng/mL	0.99 (0.44–4.8)
Serum zinc level; μg/dL	63.4 (49.7–79.8)
Positive blood cultures	97 (13.9%)
Randomised to zinc	352 (50.3%)

Data are mean (± SD), median (IQR) and n (%).

^a^Axillary temperature >37.5°C

^b^ ≥60 breaths per min for infants <2 months; ≥50 breaths per min for infants ≥2 months.

^c^ Normal values of CRP in this age group is upto 10 mg/L and for PCT is 0.6 ng/mL

TLC- Total leucocyte count, CRP- C-reactive protein, PCT-Procalcitonin, SD- Standard deviation

**Fig 1:**
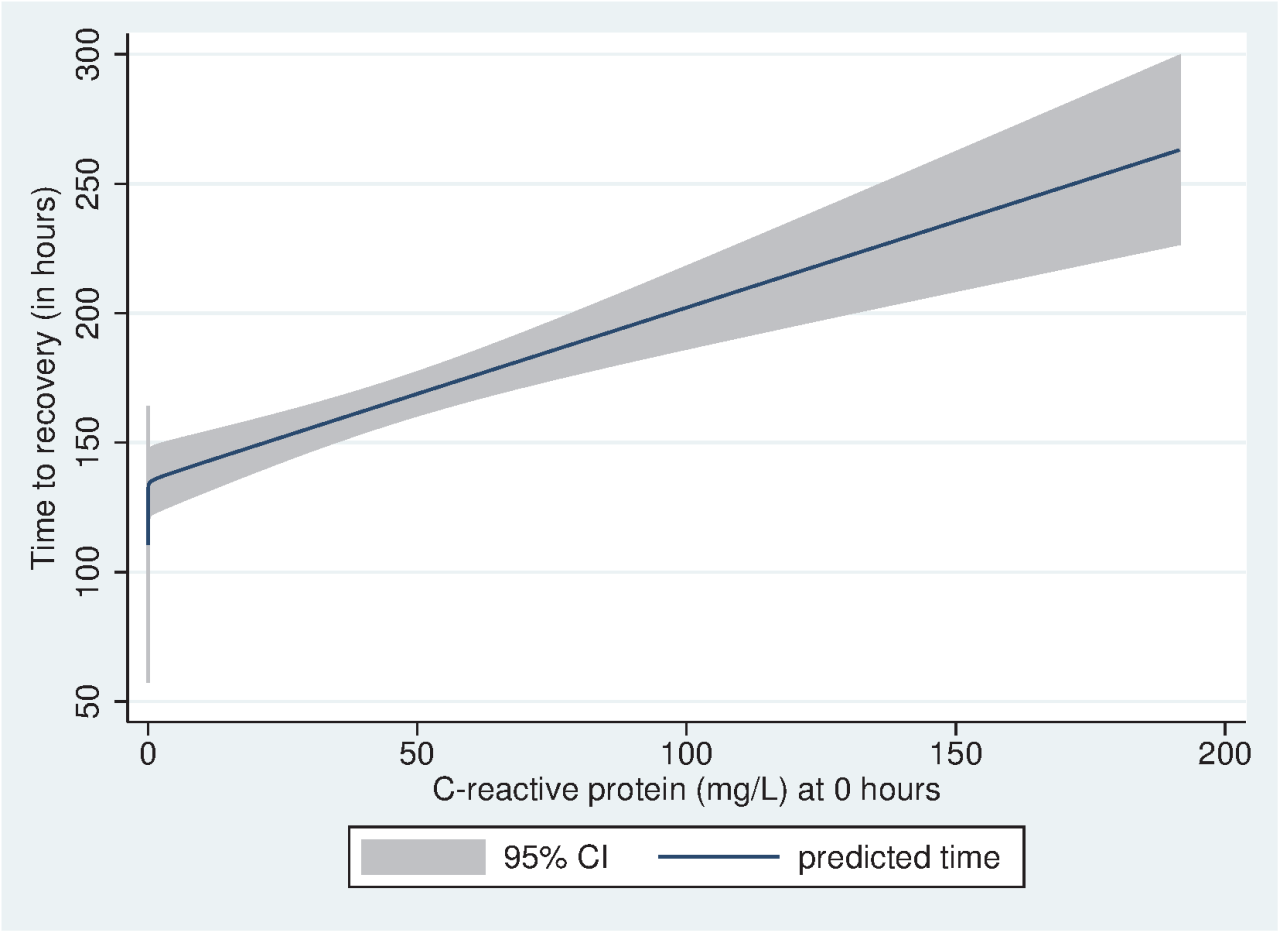
Correlation between baseline CRP level and time to recovery (in hours). Several studies in the past have used cutoffs in the range of CRP concentrations of 40 mg/L to differentiate between bacterial and non-bacterial or viral infections. [11,12]. We therefore also performed an additional analysis where we dichotomized serum CRP concentration with a cut-off of 40 mg/L. In this analysis we found that predictors for time to recovery from PSBI remained the same and infants with CRP ≥ 40 mg/L were found to have on an average 33% longer time to recovery than those with values below that.

Of the 37 baseline variables, 17 were associated with time to recovery at P-values <0.2 (Table 2) and were therefore included in the multivariable Cox proportional hazard regression model, which showed that weight for age <- 2 z score, i.e. being underweight (P = 0.047), history of formula feeding prior to the episode of illness (P = 0.003), irritability (P = 0.041), lethargy (P = 0.02) and poor sucking at breast (P = 0.001) independently predicted time to recovery (Table 3). Of the laboratory parameters, baseline CRP concentration predicted time to recovery (P<0.001). The association between CRP concentration and time to recovery was nearly linear (Fig 1).

**Table 2:** Univariable analysis using Cox regression to find variables associated with time to recovery in 7–120 day old infants with probable serious bacterial infection.

	Hazard Ratio	P-value
**Demographic details**		
Age^a^	0.99 (0.99,1.00)	0.103
Sex	0.90 (0.76,1.06)	0.210
**Clinical history**		
Irritability^a^	0.89 (0.75,1.05)	0.170
Excessive Cry	1.06 (0.90,1.25)	0.479
Diarrhea	0.99 (0.84,1.17)	0.929
Blood in stools^a^	0.50 (0.27,0.93)	0.029
Vomiting	1.11 (0.91,1.34)	0.303
Cough	1.05 (0.90,1.24)	0.518
Rapid breathing	1.01 (0.86–1.18)	0.937
Convulsions^a^	0.78 (0.58,1.04)	0.088
Edema	0.72 (0.27,1.94)	0.520
Poor oral acceptance^a^	1.17 (1.00,1.38)	0.052
History of breast milk feeding prior to the episode ^a^	1.47 (1.19,1.83)	<0.001
History of cow milk feeding prior to the episode	0.92 (0.77,1.11)	0.405
History of formula milk feeding prior to the episode ^a^	0.70 (0.56,0.88)	0.002
History of antibiotic treatment in the current episode	0.99 (0.99,1.00)	0.452
**Anthropometry**		
Weight for age less than 2z^a^	0.76 (0.63,0.90)	0.001
**Physical examination**		
Fever^b^	1.07 (0.95,1.21)	0.236
Lethargy^a^	0.75 (0.63,0.90)	0.002
Poor sucking at breast^a^	0.7 (0.60,0.83)	<0.001
Fast breathing^c^	0.98 (0.83,1.15)	0.775
Crepitations	0.99 (0.84,1.17)	0.929
Grunting	0.86 (0.62,1.20)	0.385
Wheeze	1.07 (0.85,1.35)	0.568
Cyanosis^a^	3.33 (1.07,10.4)	0.038
Capillary refill time > 3 seconds	0.72 (0.32,1.61)	0.420
Abdominal distention^a^	0.53 (0.36,0.78)	0.001
Oral thrush	0.73 (0.44,1.21)	0.223
Some dehydration	0.87 (0.66,1.14)	0.308
Bulging fontanel^a^	0.63 (0.39,1.04)	0.074
**Laboratory parameters**		
Hemoglobin^a^, g/dL	1.03 (1.01,1.06)	0.017
TLC; per μL of blood	0.99 (0.99,1.00)	0.264
Serum CRP ^a^; mg/L	0.99 (0.99,0.99)	<0.001
Serum PCT ^a^; ng/mL	0.99 (0.99,0.99)	0.005
Serum zinc; μg/dL	1.00 (0.99,1.00)	0.908
Blood culture positivity^a^	0.78 (0.61,0.98)	0.035
**Intervention**		
Zinc as treatment	0.98 (0.83–1.15)	0.778

TLC- Total leucocyte count, CRP- C-reactive protein, PCT-Procalcitonin

Hazard ratio <1 indicates slower recovery

^a^Variables included in multivariable Cox regression analysis because their P-values were <0.2.

^b^ Axillary temperature >37.5°C

c ≥60 breaths per min for infants <2 months; ≥50 breaths per min for infants ≥2 months.

**Table 3:** Baseline characteristics found in multivariable Cox proportional hazard regression to be associated with longer recovery time in 7–120 day old infants with probable serious bacterial infection.

	Hazard Ratio*	P Value
**Clinical History**		
Irritability	0.81 (0.66,0.99)	0.041
Formula feeding prior to the episode	0.67 (0.52,0.87)	0.003
**Physical examination**		
Lethargy	0.77 (0.62,0.96)	0.020
Poor sucking at breast	0.73 (0.61,0.87)	0.001
**Anthropometry**		
Weight for age less than 2z	0.84 (0.70,0.99)	0.047
**Laboratory Parameters**		
CRP (in mg/L)	0.99 (0.98,0.99)	<0.001

CRP- C reactive protein

*Hazard ratio <1 indicates slower recovery.

## Discussion

Time to recovery is an indicator of severity of PSBI and is an important parameter because length of hospital stay is relevant to family economy and health system expenditures. We identified independent clinical and laboratory predictors for time to recovery in infants admitted with PSBI to three tertiary care referral centres in India using a data set of a recently undertaken randomised controlled trial [8].

All infants in our study were less than 4 months of age and were as per World Health Organization (WHO) guidelines supposed to be exclusively breastfed [13]. Those who were on formula feeding prior to the onset of illness were found to have on an average 33% slower recovery as compared to those who were not. This is in line with the findings of a large clinical study from a similar setting, which reported several-fold increased risk for hospitalization for diarrhea and respiratory infections in exclusively formula fed infants when compared with those who were exclusively breastfed [14]. This emphasizes discouraging formula feeding and promoting exclusive breastfeeding in early infancy [15].

Underweight was an independent predictor of slower recovery in our infants with PSBI. Being underweight at six weeks of age is a known risk factor for all-cause death during the first half of infancy [16]. While studies in infancy are lacking, malnutrition has also been shown to delay recovery and prolong hospital stay [17, 18]. Undernourished babies may have decreased numbers and functional ability of CD4 and CD8 cells [19]. Reduction in the secretory IgA component and complement components could also be contributing factors [19, 20]. However, the data from these studies should be interpreted with caution as there are probably several confounders of this association such as poverty, deficiency of specific micronutrients, concurrent infections etc.

IMNCI has been validated in our setting and has been found to have 94% sensitivity and 87% specificity for identifying sick young infants of age 7–60 days [21]. Our analysis suggests irritability, lethargy, and poor sucking at breast were also predictors of time to recovery from PSBI. Thus, a subset of these patients diagnosed with severe illness using the IMNCI strategy could have more severe illness than others.

CRP is an acute phase reactant and has been used as diagnostic marker for serious bacterial infection in hospitalized febrile infants [22]. The role of CRP in diagnosing neonatal sepsis is well studied. Several studies have reported sensitivities and specificities ranging from 74 to 98% and from 71 to 94%, respectively, for either serial CRP determinations or a single determination at least 12 h after the onset of symptoms [23]. In neonatal sepsis, serial CRP measurements have been shown to be helpful in monitoring the response to treatment, to determine the duration of antibiotic therapy, and to recognize possible complications [23]. Prospective studies have shown that CRP is also a valuable marker and is more sensitive than other markers like white cell count and absolute neutrophil count in predicting serious bacterial infection in febrile infants [22,24]. Data from adults suggest that higher CRP level predicts death in patients with bacteremia and/or sepsis. It may also predict organ failure and longer intensive care stay in critically ill patients [25–27]. A similar association between elevated CRP levels and mortality has been observed in neonates hospitalized with sepsis [7]. Although extensively studied among neonates and adults with sepsis, there is a paucity of data on its utility as a prognostic marker in older infants with severe infections. We found that CRP is an independent predictor of time to recovery in infants with PSBI and is nearly linearly associated with duration of hospitalization. This important finding could be the basis for recommending CRP as a simple tool for predicting the severity of illness in infants admitted with features of PSBI and help in decision making for clinical management.

Our study has some limitations. We only included children with symptoms of possible serious bacterial infection and elevated serum CRP concentration. Thus, our findings might not be representative of all the infants with possible serious bacterial infection. In addition to the factors mentioned above, birth weight could also be associated with time to recovery. However, we did not have data on this variable. Weight gain per day is dependent on baseline weight and is usually assessed in weight/kg/day and should be used if weight gain is the primary objective. In our study where weight gain was part of a composite outcome, we felt that after recovering from an acute illness, the weight of infants would either be stable or increasing. As long as the infant had some weight gain (atleast 10 gram/day) and he/she had no symptoms of PSBI and was on oral feeding, this could be assumed to be a sign of recovery

Nonetheless, ours is the first study evaluating predictors of time to recovery in young infants with PSBI and is based on a large dataset of 700 infants with PSBI with a very close follow-up from admission till recovery. Further studies to identify simple predictors of time to recovery in infants diagnosed with possible serious bacterial infection based on clinical history and signs/symptoms used in IMNCI strategy alone would enable clinicians to easily use them for clinical decision making at primary and secondary health care settings. Although any infant with sepsis needs to be managed aggressively, in resource limited settings such as ours, many a times infants with PSBI are not referred to higher centres or not managed with proper treatment such as early initiation of antibiotics. All the infants included in our study had probable serious bacterial infection (which could very well suggest sepsis) but a subset of clinical and laboratory markers in these infants suggested prolonged recovery. This subgroup of infants needs even more attention including prompt referral to higher centres and this study thus helps in triaging.

Also, it is of prognostic information to clinicians taking care of these infants and infant’s family members that a subset of these infants might have longer recovery time as these have economic as well as social implications on the family especially in resource limited settings.

## Conclusion

We identified predictors for time to recovery in young infants with PSBI that could be used for early identification of infants at risk for longer recovery time and prompt referral to higher level centers and aggressive management could be helpful in these infants.

## References

[1] Liu L, Oza S, Hogan D, Perin J, Rudan I, Lawn JE, et al (2015) Global, regional, and national causes of child mortality in 2000–13, with projections to inform post-2015 priorities: an updated systematic analysis. Lancet 385:430–40. 10.1016/S0140-6736(14)61698-6 25280870

[2] UN Inter-agency Group for Child Mortality Estimation (2013) Levels and Trends in Child Mortality. Available: http://www.unicef.org/media/files/2013_IGME_child_mortality_Report.pdf. Accessed 25 February 2015.

[3] The Million Death Study Collaborators, Bassani DG, Kumar R, Awasthi S, Morris SK, Paul VK, et al (2010) Causes of neonatal and child mortality in India: a nationally representative mortality survey. Lancet 376: 1853–60. 10.1016/S0140-6736(10)61461-4 21075444 PMC3042727

[4] Weber MW, Carlin JB, Gatchalian S Lehmann D, Muhe L, Mulholland EK, et al (2003) Predictors of neonatal sepsis in developing countries. Pediatr Infect Dis J 22: 711–7 12913772 10.1097/01.inf.0000078163.80807.88

[5] Metsvaht T, Pisarev H, Ilmoja ML, Parm U, Maipuu L, Merila M, et al (2009) Clinical parameters predicting failure of empirical antibacterial therapy in early onset neonatal sepsis, identified by classification and regression tree analysis. BMC Pediatr 9: 72 10.1186/1471-2431-9-72 19930706 PMC2789707

[6] Venkataseshan S, Dutta S, Ahluwalia J, Narang A (2007) Low plasma protein C values predict mortality in low birth weight neonates with septicemia. Pediatr Infect Dis J 26: 684–8. 17848878 10.1097/INF.0b013e3180f616f0

[7] Suri M, Thirupuram S, Sharma VK (1991) Diagnostic and prognostic utility of C-reactive protein, alpha-1-antitrypsin and alpha-2-macroglobulin in neonatal sepsis: a comparative account. Indian Pediatr 28: 1159–64. 1724658

[8] Bhatnagar S, Wadhwa N, Aneja S, Lodha R, Kabra SK, Natchu UC, et al (2012) Zinc as adjunct treatment in infants aged between 7 and 120 days with probable serious bacterial infection: a randomised, double-blind, placebo-controlled trial. Lancet 379: 2072–8. 10.1016/S0140-6736(12)60477-2 22656335

[9] Ministry of Health and Family Welfare Government of India (2003) Integrated management of neonatal and childhood illness: physician chart booklet. Available: http://www.unicef.org/india/Chart_Booklet.pdf. Accessed March 1 2012.

[10] National Center for Health Statistics (2000) Individual growth charts. Available: http://www.cdc.gov/growthcharts/charts.htm. Accessed March1 2012.

[11] Coles CL, Bose A, Moses PD, Mathew L, Agarwal I, Mammen T, et al (2007) Infectious etiology modifies the treatment effect of zinc in severe pneumonia. Am J Clin Nutr. 86:397–403. 17684211 10.1093/ajcn/86.2.397

[12] Korppi M, Kroger L (1993) C-reactive protein in viral and bacterial respiratory infection in children. Scand J Infect Dis 25:207–13. 8511515 10.3109/00365549309008486

[13] World Health Organization (2002) Infant and young child nutrition. Available: http://apps.who.int/gb/archive/pdf_files/WHA55/ea5515.pdf. Accessed July 2 2014.

[14] Hengstermann S, Mantaring JB 3rd, Sobel HL, Borja VE, Basilio J, Iellamo AD, et al (2010) Formula feeding is associated with increased hospital admissions due to infections among infants younger than 6 months in Manila, Philippines. J Hum Lact 26: 19–25. 10.1177/0890334409344078 19759351

[15] Bhandari N, Bahl R, Mazumdar S, Martines J, Black RE, Bhan MK, et al (2003) Effect of community-based promotion of exclusive breastfeeding on diarrhoeal illness and growth: a cluster randomised controlled trial. Lancet 361: 1418–23. 12727395 10.1016/S0140-6736(03)13134-0

[16] Vesel L, Bahl R, Martines J, Penny M, Bhandari N, Kirkwood BR, et al (2010) Use of new World Health Organization child growth standards to assess how infant malnutrition relates to breastfeeding and mortality. Bull World Health Organ 88: 39–48. 10.2471/BLT.08.057901 20428352 PMC2802434

[17] Lim SL, Ong KC, Chan YH, Loke WC, Ferguson M, Daniels L (2012) Malnutrition and its impact on cost of hospitalization, length of stay, readmission and 3-year mortality. Clin Nutr. 31: 345–50. 10.1016/j.clnu.2011.11.001 22122869

[18] Kyle UG, Coss-Bu JA (2010) Nutritional assessment and length of hospital stay. CMAJ 182: 1831–2. 10.1503/cmaj.101256 20940231 PMC2988528

[19] Chandra RK (1997) Nutrition and the immune system: an introduction. Am J Clin Nutr 66: 460S–63S. 9250133 10.1093/ajcn/66.2.460S

[20] Sirisinha S, Suskind R, Edelman R, Asvapaka C, Olson RE (1975) Secretory and serum IgA in children with protein-calorie malnutrition. Pediatrics 55: 166–70. 4213370 10.1007/978-1-4613-4550-3_46

[21] Kaur S, Singh V, Dutta AK, Chandra J (2011) Validation of IMNCI algorithm for young infants (0–2 months) in India. Indian Pediatr 48: 955–60. 21555803 10.1007/s13312-011-0155-1

[22] Bilavsky E, Yarden-Bilavsky H, Ashkenazi S, Amir J (2009) C-reactive protein as a marker of serious bacterial infections in hospitalized febrile infants. Acta Paediatr 98: 1776–80. 10.1111/j.1651-2227.2009.01469 19664100

[23] Hofer N, Zacharias E, Müller W, Resch B (2012) An update on the use of C-reactive protein in early-onset neonatal sepsis: current insights and new tasks. Neonatology 102:25–36. 10.1159/000336629 22507868

[24] Andreola B, Bressan S, Callegaro S, Liverani A, Plebani M, Da Dalt L (2007) Procalcitonin and C-reactive protein as diagnostic markers of severe bacterial infections in febrile infants and children in the emergency department. Pediatr Infect Dis J 26:672–7 17848876 10.1097/INF.0b013e31806215e3

[25] Devran O, Karakurt Z, Adıgüzel N, Güngör G, Moçin OY, Balcı MK, et al (2012) C-reactive protein as a predictor of mortality in patients affected with severe sepsis in intensive care unit. Multidiscip Respir Med 7: 47 10.1186/2049-6958-7-47 23171626 PMC3529702

[26] Lobo SM, Lobo FR, Bota DP, Lopes-Ferreira F, Soliman HM, Mélot C, et al (2003) C-reactive protein levels correlate with mortality and organ failure in critically ill patients. Chest 123: 2043–9. 12796187 10.1378/chest.123.6.2043

[27] Gradel KO, Thomsen RW, Lundbye-Christensen S, Nielsen H, Schønheyder HC (2011) Baseline C-reactive protein level as a predictor of mortality in bacteaemia patients: a population-based cohort study. Clin Microbiol Infect 17: 627–32. 10.1111/j.1469-0691.2010.03284.x 20545964

